# Supplementary material for: Neural speech restoration at the cocktail party: Auditory cortex recovers masked speech of both attended and ignored speakers
Source: PLoS Biol. 2020 Oct 22;18(10):e3000883. doi: 10.1371/journal.pbio.3000883 (PMC7644085; doi:10.1371/journal.pbio.3000883)
Supplement: S1 Simulations — TRF, temporal response function. (PDF) [file pbio.3000883.s001.pdf]

Simulations to assess TRF cross-contamination

Simulations were used to determine whether some of the results presented in the main paper could be signal processing artifacts. One main finding in the MEG study concerns TRFs to onsets in the ignored speech source, above and beyond onsets in the acoustic mixture (Fig 3). This deserves special care because these two predictor variables are not orthogonal. Specifically, there is a concern that a true response to the mixture might cause a spurious response to the ignored source. Simulations designed to address this concern were run in order to determine whether such cross-contamination might be seen (Fig S1A). Additional simulations were performed to look for unexpected interactions of TRF latency estimates in different predictors (Figs S1B and S1C).

Methods

Simulations were performed using the same stimuli used in the MEG experiment, with the envelopes and onsets downsampled to 100 Hz. Simulated TRFs were manually pre-determined, using a combination of Gaussian windows, to resemble experimental TRFs. The first simulation generated responses to the mixture and the attended source of envelopes and onsets, respectively (red lines in Fig S1A). No TRFs were assigned to the ignored source, because detecting spurious TRFs in the ignored source was the aim of this simulation. The simulation encompassed 40 simulated subjects. For each subject, attention alternated between the female and male stimulus for the stimulus pairs 1-4 (order counterbalanced as in the MEG experiment). Each stimulus pair was used only once, i.e., each simulation used 4 minutes of data. The response was simulated by convolving each of the 256 bands of each predictor with the TRF corresponding to the predictor, summing all responses, and adding pink noise based on the Voss-McCartney algorithm, with a signal to noise ratio (SNR) of 1:10 (i.e., -10 dB SNR). TRFs were then reconstructed with the same algorithm as in the main experiment, and the same predictors binned into 8 frequency bins. Simulations S1B and S1C involved only slight modifications of the simulated responses which are described below.

Results

The first simulation was designed to look for spurious responses to the ignored speech. Fig S1A displays the simulated TRFs (thick red lines) overlaid on the STRFs estimated in the simulation (blue/yellow lines with standard error). Onset TRFs exhibit no notable spurious response to the ignored speaker, suggesting that leakage from onset responses to the mixture is unlikely to be the source of the results obtained in the MEG study.

Simulations S1B and S1C were designed to assess latency shifts in the estimated TRFs. The results from the experimental MEG responses, discussed in the main paper, indicated a systematic difference in response function latencies between the onset mixture representation and the individual streams. Thus, a concern arose as to whether peak latencies to those representations systematically affect each other. The simulations shown in Fig S1B were identical to S1A, except that the early onset peaks were somewhat narrower, and the latency of the attended stream response was systematically shifted to be earlier than, simultaneous with or later than the mixture

response (indicated by the vertical dotted lines). Results indicate that each peak might be slightly mislocalized towards the latency of the other peak. This indicates that if any bias were present in the estimation, it would have worked against the results observed in the experimental responses. The simulations in Fig S1C repeated this test but also assessing the influence from a response to the ignored talker. The simulations were identical to those in Fig S1B, but added a response to the ignored talker, consisting of the first peak of the attended talker. Results again show a slight tendency for the mixture peak to be mislocalized in time towards the attended/ignored speaker peaks. In addition, they suggest that in the presence of identical responses to the two speakers, the mixture peak amplitude might be somewhat overestimated, which can be understood as a consequence of the mixture predictor being correlated with (though not identical to) a sum of the two speakers.

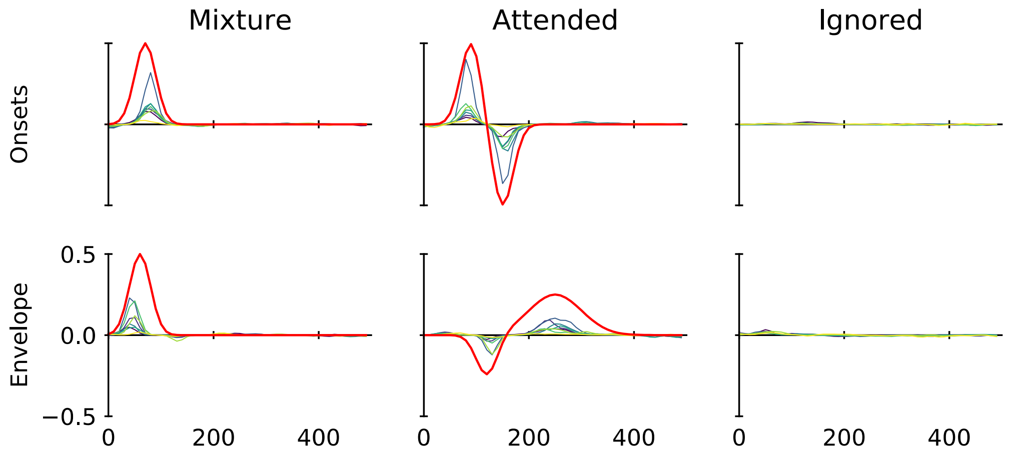

Fig S1A. **Simulation without response to ignored speech.** Simulation with conditions selected to approximate results obtained in the MEG study shown in Fig 3. Solid red lines indicate the simulated TRFs; yellow/blue lines show the STRFs reconstructed with methods analogous to the MEG analysis. Note the absence of spurious responses to ignored onsets, indicating that the algorithm used here is robust against false positives regarding ignored onsets. Shading indicates within-subject standard error of the mean. Simulated and estimated TRFs are at different scale. Data in S5 Data.

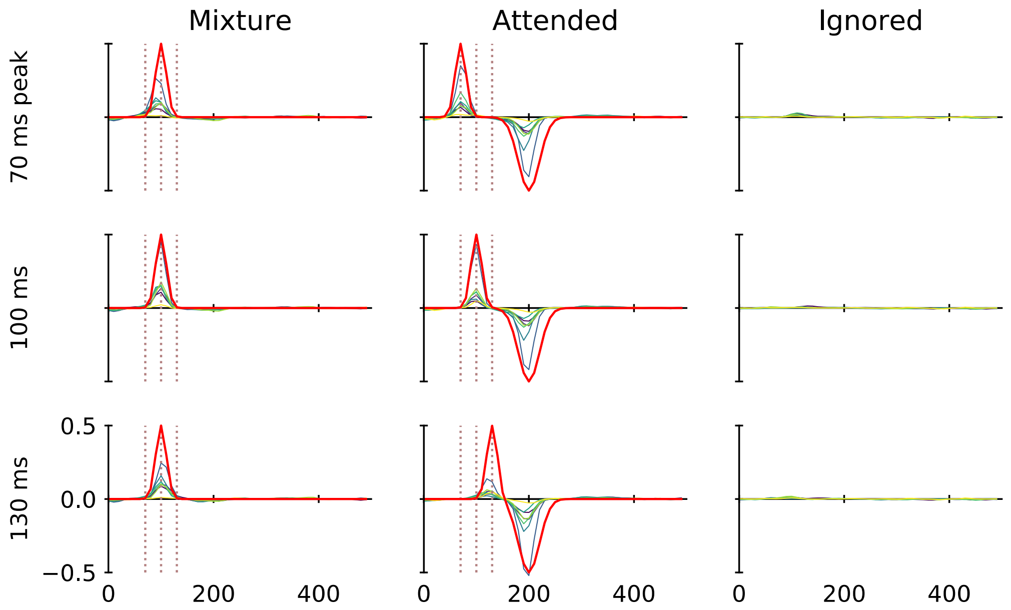

Fig S1B. **Simulations with different peak latencies.** Three simulations in which the latency of the attended onsets peak was systematically varied (dotted vertical lines). Simulations were performed to test whether a delay in the response to the attended speech, as observed in the MEG experiment, could be due to how the two responses combine. In each simulation, estimated peak times are slightly biased *towards* the actual peak of the other representation, suggesting that MEG results are not due to such an artifact. Envelopes were included in the simulations as in Fig S1A, but responses are not shown here. Data in S5 Data.

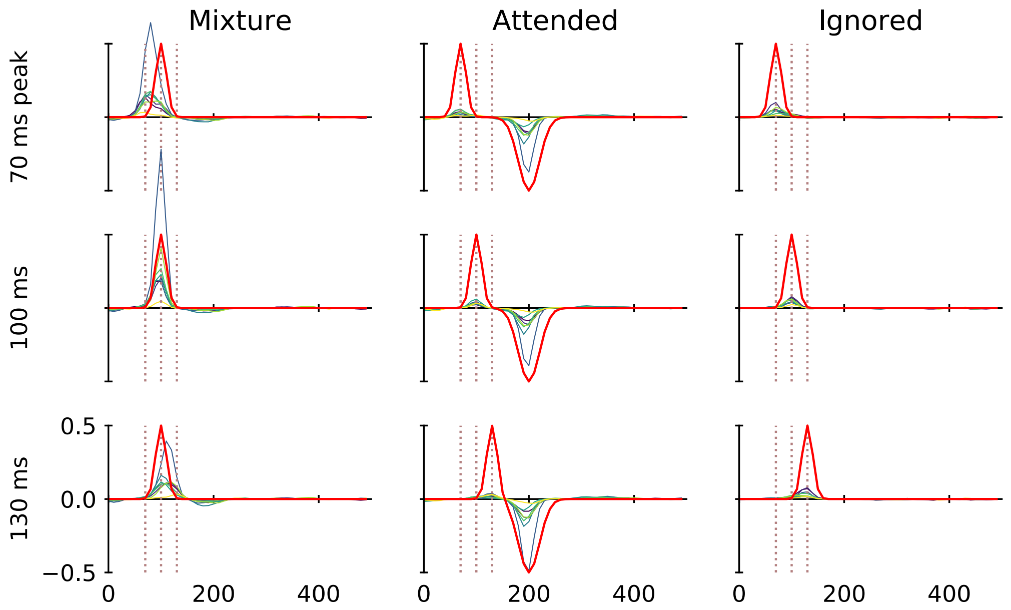

Fig S1C. **Simulation with different peak latencies, including ignored speaker**. Three simulations, analogous to Fig S1B except for including a simulated response to the ignored speaker. Results again show a slight bias in the mixture TRF *towards* the speaker TRFs, and do not suggest a bias that would cause spurious latency differences looking as those observed in the MEG results. Data in S5 Data.
