# Supplementary figures and images for: Neural speech restoration at the cocktail party: Auditory cortex recovers masked speech of both attended and ignored speakers
Source: PLoS Biol. 2020 Oct 22;18(10):e3000883. doi: 10.1371/journal.pbio.3000883 (PMC7644085; doi:10.1371/journal.pbio.3000883)

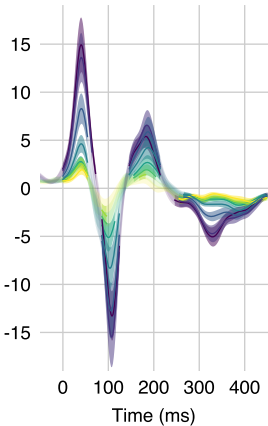

Supplement: S1 Fig — Spectrotemporal response function to the envelope spectrogram, when estimated without considering onsets. All other details are analogous to Fig 2E. Data in S4 Data. (PDF) [file pbio.3000883.s002.pdf]
