# Supplementary material for: Neural speech restoration at the cocktail party: Auditory cortex recovers masked speech of both attended and ignored speakers
Source: PLoS Biol. 2020 Oct 22;18(10):e3000883. doi: 10.1371/journal.pbio.3000883 (PMC7644085; doi:10.1371/journal.pbio.3000883)
Supplement: Attachment — Submitted filename: R2-Response v8 clean.docx [file pbio.3000883.s008.docx]

Dear Dr. Roberts and Reviewers,

We thank the reviewers for their insightful comments and suggestions, and for the opportunity to revise the manuscript accordingly. The reviewers raised several issues that led us to make important modifications and additions to the analyses, and we believe that these modifications improve the analysis and presentation of the results.

Perhaps the most significant change concerns a modification in the statistical analysis. Our original analysis tested model fit improvements from different predictors against shuffled versions of the same predictors. While addressing a concern for a better presentation of these statistical results, we made some observations which led us to change the model comparison approach (see reviewer #1, issue #1). Instead of using shuffled models to control for overfitting, we now use cross-validation. This procedure turned out to be more powerful and lead to some changes in the results, which are reflected in the updated figures. Most notably, the new tests are powerful enough to also distinguish envelope representations of the mixture and the two speakers. The main results and conclusions remain unchanged.

We would also like to highlight the new Figure 5. We have added this figure because of several comments regarding the clarity of the conclusions. Figure 5 captures the central implications of the new results in an explicit and (hopefully) intuitive way.

Below we address each issue raised by the reviewers in turn.

Sincerely,

Christian Brodbeck, Alex Jiao, L. Elliot Hong & Jonathan Z. Simon

# Reviewer #1:

Brodbeck and colleagues present an experiment and its results on the neural processing of two-talker auditory scenes as measured with MEG. Participants were presented with two audio-books and asked to focus on one of them. In a first analysis, using the same analysis framework throughout the manuscript, the authors present support of using onset predictors in addition to envelope predictors. Next, modelling MEG signals with onset and envelope predictors at different stimulus frequencies for the mixed, attended and unattended speech, the authors show TRF models showing similarities and differences between coefficients of the onset and envelope pointing out differences in the shape and latency differences of high coefficients (peaks) between attended and ignored. In a final step, the stimuli are differently described; this time in terms of overt and masked onsets, separately for attended and ignored speech. Here, the authors find a longer latency for overt vs. masked onsets

concerning a first peak in coefficients and a second peak of coefficients in attended speech that is absent for both overt and masked onsets for ignored speech. The authors suggest that this latency difference indicates active unmasking processes (similar to "filling in") in auditory cortex.

While the study is well designed and the analyses seem to be carefully performed, I have some concerns regarding the presentation of the study as well as the methodology. Main and minor points are listed below.

Major points:

1.	The analysis focuses mostly on the TRF models and does a good job in presenting the spread across participants in figures 2 to 4. However, such a strong (visual) focus on model coefficients underlines these outcomes while model performance seems "second stage". However, the focus of the effects should be on the model performance which provides a more convincing argument in this type of analyses as model coefficients are difficult to interpret (e.g., being inherently multivariate). Thus, it would be beneficial to provide figures on model performance as well (boxplot/violin plots + single participant indicators similar to 3D, 4C). Similarly, an analysis restricted to specific delay values (e.g., first, second, later peaks; similar to e.g. Puvvada and Simon, 2017) would make a better argument concerning their presence/absence. For latency changes, TRF models might be a good way pursue if one doesn't want to use shifting- window-like or single delay analyses.

We strongly agree with the reviewer that model comparisons provide crucial empirical support. In order to increase focus on the model performance in the updated manuscript, we added plots showing individual subject model fit improvements for all predictors. These plots show values that might be slightly different from the group level model tests, which also incorporated spatial consistency between subjects (see next answer). However, they do provide an overall informative picture of subject-specific outcomes.

When working on figures for individual subject model performance we noticed a side-effect of shuffled models. When an unshuffled predictor is correlated with other predictors, then shuffling that individual predictor reduces its correlation with the other predictors. This increases the effective number of degrees of freedom in the model, which in turn allows for more overfitting in shuffled models. When this occurs, the shuffled models become an overly conservative baseline, decreasing the statistical power of the test. This was tolerable for the spatial cluster-based model tests, which benefit from the spatial patterns of the effects (see next answer). However, when averaging model fits in the whole ROI, this led to estimates of individual subject fits that did not adequately reflect the spatially sensitive cluster-based group level tests. This could even manifest as the shuffled models explaining the data *better* than the true models in regions well outside of auditory areas. In order to avoid this problem, we re-evaluated all models using true cross-validation, which allowed us to avoid all use of shuffled models. It turned out that the significance tests based on cross-validation were indeed more powerful and revealed some additional significant predictors.

We did not implement model tests for specific delay values because of difficulties in formulating the tests feasibly. First, we do not have priors concerning delay values for the different representations. Hence, we would have to define time windows based on looking at TRFs first, which would make the model test a post-hoc analysis. On top of that, isolating a specific latency in a model is difficult due to autocorrelation in acoustic speech signals. TRFs account for autocorrelation in that the different latencies compete for explaining the same data: by explaining variance with the latency bins that are able to *best* explain this variance, other latency bins are then prevented from being influenced by the same variance component through autocorrelation. A model estimated on a subset of latencies does not have that mechanism and would be more prone to confounding responses at latencies outside its range due to autocorrelations.

In the light of these considerations, TRFs seem to us to be a good (though not perfect) compromise. In addition to minimizing the influence of autocorrelation as described, TRFs are also generally interpretable because they can be compared to evoked responses, and they tend to be consistent across different experiments, which facilitates comparison with other published results.

2.	The statistical testing and results reporting was unclear. This concerned both model performance comparisons as well as statistics on TRF coefficients. For example, I could not follow why, for comparing model performances from filtered MEG data optimized to sources in AC, TFCE is required. Why is a cluster analysis needed at this stage when the model performance is a single r-value that is compared between different models or with respect to a baseline? Also, what does t_max mean in this context? I see the use for the analysis of TRF coefficients but I don't see how analyses of model performance could benefit from it. In addition, the multiple comparison correction for the model coefficients remains unclear. I believe that the results are corrected as there is an indication in the final paragraph of the methods but that was not enough to fully follow the approach.

We agree that there was not enough clarity in our descriptions. When testing models in the STG/HG ROI, the models were still fit to all dipoles *in that region* separately, and evaluated with a spatial cluster-based (TFCE) test. The signal from the different dipoles were only combined for STRF analysis, primarily to make possible an intuitive visualization. We have now clarified this when reporting results (line 204) and in the Methods section (line 632 ff.). t_max is the maximum value of the t-statistic that appeared in the significant cluster; this value can be used for effect size comparisons (we have added a corresponding description to the Methods on line 630 ff.).

3.	While I like and agree with the conclusions drawn especially from the overt vs masked onset comparisons, I don't follow why this manuscript's title (and to a lesser degree the abstract and discussion) focuses to such a large extent on background speech processing rather than the masking results. In my opinion, the effects and conclusions on overt vs. masked onsets (amplitude and latency differences) constitute the main content being novel to the field while the absence of the second negative peak is interesting (it lends support to the hierarchical processing accounts discussed by the authors) seems rather like a strong but secondary result/conclusion.

We agree and have updated the abstract and discussion to add more focus on the representations of overt vs. masked onsets (lines 29 and 345 ff.). We have also changed the title to better reflect the importance of the various findings. This is also highlighted in the new Figure 5.

4.	One of the main points of this paper is the comparison of onset and envelope descriptions of sound stimuli to explain MEG activity. While I agree with the conclusions and like how carefully these distinctions were analyzed and interpreted, I want to remind the authors that studies have already applied onset descriptions for the analysis of continuous sounds in similar analysis frameworks which is not mentioned in this study (e.g., Petersen et al., 2017, JNeurophysiol; Fiedler et al., 2017, JNeurEng, Fiedler et al., 2018, NeuroImage; Hausfeld et al., 2018, NeuroImage; and maybe more). Still, the explicit comparison and quantification of including onset features provided in this study provides a novel element.

Those references are indeed important, and we have now updated the Introduction section on onset responses to more clearly state that prior studies have used, and benefited from, similar onset representations (line 90).

Minor points:

5.	Related to point 2: figures 2D, 3B and 4B include grey-scale indicators of significance for specific delays, which are markers for significantly non-zero coefficients in any frequency band (l. 147/8). Were these already corrected for multiple comparisons (e.g., by removing small clusters not surviving a cluster-based thresholding) and was this performed across 2 dimensions (frequency and delay) or across the delays? While there is a lot of information conveyed in these graphs, simply indicating any significance across frequencies is a big simplification. How about adjusting the height of the significance bar according to the number of significant frequency bins? Another small point is that at the moment it is unclear which p-values are indicated: max, min, an average of some sort.

We agree that our original choices made for simplifying those figures were flawed, and we greatly appreciate the suggestions. We have now updated the figures to indicate significance in a band-specific way: significant elements are printed with saturated colors, whereas non-significant elements are desaturated.

6.	Ll. 185-188 and Fig 3C: This was difficult to follow.

Agreed. We have now removed this portion because it is no longer relevant to the updated results with cross-validation.

7.	It is suggested masked onsets of both attended and unattended speech undergo active processing to be "un-mask". Especially for unattended sounds this might be interesting for further investigation as it remains unclear with these data whether this unmasking works on single speech sources or more generally on the unattended sounds as a whole (representing attended vs all other sounds). Could you please comment on that in your response?

We agree that this is an open and interesting question. The experiments to address this are not necessarily easy. One way would be to recreate the three-equally-loud-speakers (one attended, two background) experiment of Puvvada & Simon (2017) and analyze the background speaker pair as a single background source. That was a tricky experiment, however. Another might be to mix one (attended) speaker with (somewhat lower intensity) babble and analyze the babble. But extended selective attended listening to only one speaker (not rotating among two or three) may engage different mechanisms for speaker segregation than the ones engaged here, as well as small vs. large numbers of speakers contributing to the babble. We have added a corresponding note in the Discussion (line 490 ff.).

8.	The figures are difficult to read given their size, please make them bigger or maybe split them up such that single panels have more space.

This is partially a problem of the submitted pdf generated from figures embedded in Word: the figures are designed to use the entire page width. In the context of a typeset journal page, the figure typeface should be comparable in size to the journal typeface. Nevertheless, we do appreciate the issue and we have re-organized some of the figures for greater ease in reading.

9.	The color differences between minus and plus peaks are impossible to distinguish (maybe it was the print out but the colors seems to be very similar). Are the color differences between positive and negative peaks necessary? For the line graphs these peaks are easily discernible which also holds for the single participants markers in Fig. 3C

We agree that a color distinction between negative and positive peaks is not necessary. In the updated figures we use a more intuitive color scheme, with one color per predictor.

# Reviewer #2:

This is an interesting paper that uses previously published MEG data from humans listening to connected speech, either as a single talker or two simultaneous talkers (one male, one female) with participants attending to one and ignoring the other. The study reports a difference in how "masked" onsets in speech are represented in the human auditory cortex, relative to onsets that are not masked by the competing talker. This difference is observed both for attended and unattended talkers. The authors suggest in the paper (including in the title) that this difference reflects an "active" process, and that the longer latency observed for the masked responses may reflect this active processing (or recovery) of the masked speech.

Although the premise is interesting, I do not believe that the authors have demonstrated what they claim. In particular the conclusions are based on linear systems analysis, which is applied to a highly nonlinear system. This approach of assuming linearity is almost universally adopted and has been a very useful first approximation, in part because speech is quite sparse, and so even mixtures of two talkers will having relatively limited spectrotemporal overlap. Nevertheless, the limitations of a linear-systems approximation to a nonlinear (and time-variant) system should not be overlooked. An important limitation is that a linear system assumes perfect superposition, whereas neural recordings do not demonstrate this. Indeed, the simplest auditory responses, from the Wave I, reflecting auditory-nerve responses, through brainstem responses (e.g., Wave V), to early cortical responses, all show changes in the morphology of the responses (amplitude and latency) based on the signal-to-noise ratio, ie, the degree to which a stimulus is masked. By this reasoning, it would be expected that partially masked sounds would produce smaller-amplitude and longer-latency responses, just as reported by the authors, not because of any active stream segregation, but because neural responses to partially masked sounds typically have smaller responses and longer latencies. Perhaps I am missing something important, which answers this criticism, and for this reason I have recommended revision rather than initial rejection. In any case, this seems an obvious alternative explanation, so it is not clear why the authors haven't addressed it, if only to rule it out.

We agree that this is a topic that should be addressed. We have now added a discussion of the relationship between masking and response latency (section *Delayed response to masked onsets*, line 345 ff.). More importantly, we have also implemented an additional model to test for an alternative explanation of our finding based on a non-linear response to the mixture (same section).

Specific comments

The onset detection used by the authors seems to be based on a neural model. This needs a little more explanation, as well as more detail. For instance, what proportion of onsets overall were deemed "masked"? Also the measure itself (Max(0,(Max(attended,ignored) - mixture) could do with a little more explanation. Are the units in terms of the model's neural response? Does this measure have any influence on the size of the MEG responses reported in the figures?

We have added an explanation of the onset detector model to the legend of Figure 2. We have also added more details to the explanations of the masked onset model, including the proportion assigned to masked and overt onsets (lines 276 ff., 580 ff.). Predictors and responses were all normalized for STRF estimation. We have also added this information to the Methods section (line 651 ff.).

Line 319. Early selective attention. The authors suggest that the masked responses show earlier manifestations of attention than the overt responses. But according to Fig. 4B, both the unmasked (overt) and the masked responses start to show significant effects of attention prior to 100 ms, and the difference seems to be more in the sign than in the magnitude of the difference. This needs to be clarified or reframed.

We agree that this is an important consideration for the interpretation of the results and have added a new comment to draw attention to this distinction (line 342).

Line 439. Add ", respectively" after "Chapters 3 and 8" so that it's clear that the male read 3 and the female read 8.

Added.

Line 442. How was loudness matched, and what was the approximate sound pressure level of the two talkers? (Something more exact than "Comfortably loud")

The loudness was matched not by acoustic power but so that young, normal hearing, native English listeners reported equal ease in attending to either speaker. The sound pressure level was approximately 70 dB SPL. We have now added this information to Methods (lines 531, 538).

Line 507. Typo: "am" should be "an".

Fixed.

# Reviewer #3:

This manuscript presents research aimed at investigating the representation of attented and, especially, unattended speech in the auditory cortex. The authors ask subjects to attend to one of two concurrently stories at the same time while they record their MEG. Then they examine how well they can predict MEG, source localized to auditory cortex, from two different representations of the speech stimuli. One representation is a gammatone spectrogram of the stimulus (essentially eight envelopes corresponding to the energy in eight frequency bands). The second is a spectrogram of the acoustic onsets of the speech, again in eight bands, and derived using an auditory onset model. The goal is to test if early auditory cortex either conducts a fairly straightforward and stable spectrotemporal analysis of all input or carries out a more active process to that involves representing speech features from attended and unattended streams in a dissociable way. They reason that including the acoustic onset representations should allow them to answer this question because of the importance of onsets to auditory scene analysis and also because, analytically, the onsets in a mixture of two speech streams is not very predictable from the mixture of the onsets of the two streams. They report that… and conclude that…

Overall I thought this was a very interesting manuscript with a well conducted experiment and a sensible and sophisticated data analysis framework. I did have a few queries and comments for the authors though.

Main comments:

1)	My first comment just speaks to the idea of making the rationale for the approach a bit more reader friendly in the introduction. I have to admit it took me two reads through the introduction for me to start to become comfortable with the ideas underlying the strategy. So, can I first clarify: the authors use the onset representation for two reasons: a) because of their importance for ASA, and b) because of this property that "the proportion of the variability in the mixture representations that cannot be predicted from the two sources is small for the envelopes, but substantially larger for the onsets". Is that accurate?

This characterization is correct – Initially, we used onsets mainly because of their relevance for comprehension and auditory object perception/stream segregation. While working with these representations, we then realized that onsets and envelopes differed in how well we were able to distinguish the foreground and background from the mixture. We explained this as a result of the difference in the interference patterns.

The updated results using cross-validation do not actually show this same pattern anymore, however (we can now distinguish foreground and background from the mixture using envelopes). Consequently, this discussion seems less relevant. As it is, as you pointed out, also hard to follow, so we decided to remove it from the manuscript.

If so, I wondered about giving readers a slightly more intuitive description of this. The way I have phrased it above is that "the onsets in a mixture of two speech streams is not very predictable from the mixture of the onsets of the two streams", at least less than for the envelope. Am I understanding what you are saying?

Yes, this is correct. We believe the new, more streamlined, Introduction should be clearer with regard to this.

Also, I was confused about how this links with the idea that "With acoustic onset features, interference is more pronounced because ongoing acoustic energy in one source can hide an increase in another source (see below for empirical verification of this claim)." Is this making the same point? It didn't seem so on first reading. And it was not at all obvious to me - I would have felt that envelopes were more likely to interfere with each other because they are more continuous. You say you empirically verify this below - is that in Figure 3C).

We apologize for the confusion. Maybe a better characterization would have been that onsets experience *negative* interference, while envelopes combine mostly additively. This can be seen in the spectrograms in Figure 3: the mixture *envelope* representation largely looks like the sum of the two stream envelope representations. The mixture *onset* representation, on the other hand, has several features that have a *lower* amplitude than the corresponding feature in the stream. We have added this explanation to the main text (line 255 ff.).

Anyway, the reason I am commenting on this, is that when I got to the results I just had this nagging feeling that the delay I was seeing in the STRFs for the individual sources might just be a signal processing glitch. Does the supplementary figure answer that specific concern? Or is that only to provide evidence that the ignored speech source contribution cannot be explained from the mixture. It feels like only the latter to me.

The simulation was originally implemented for the latter only, but we appreciate this concern. We have now added another set of simulations to specifically explore the question of whether peak latencies might be systematically distorted (Figure S2).

2)	Related to the previous point, is it also fair to say that the current analysis basically seeks to overturn the claims made in the Puvvada & Simon (2017) paper - based on an analysis that is more sensitive to the neurophysiological responses to the individual speech streams?

We think it would be fair to say this, though the new results can also be viewed as an elaboration of the older results with more detail. The earlier paper asked either-or questions, testing whether responses are, overall, better described as representations of segregated streams or as a mixture. The new investigation allows for more detailed questions about different representations simultaneously present in the responses. The new results confirm the earlier results, insofar as they show that early responses are dominated by the mixture (rather than pre-segregated streams), but in addition to that, they also show new evidence of partially segregated representations, which we interpret as evidence for an early stage of the cortical segregation processes. We have added new text to clarify this (lines 454 ff.).

3)	Given recent (cited) work on the different effects of attention in HG vs STG (O'Sullivan et al., 2019), I also wondered about the auditory localization used in the present study. I think I understand that the present study does not contradict the O'Sullivan paper. It merely suggests that both attended and unattended speech are separably represented in early auditory cortex, not that both are modulated strongly by attention in early auditory cortex. Nonetheless, I wondered about how confident we can be that we are really looking early auditory cortex not contamination by strong attention effects from, for example, STG. I guess it all comes down to the latency of the effects you are seeing? The source onset STRFs in Figure 3B show strong attentional modulations of a later component around 150 ms - might this be STG? And so we are safe to assume everything before 100 is from a "lower" cortical area than STG?

Answering localization questions with MEG on such a fine grain is tricky. Our results show dominant vertical currents at each stage. However, those are also the currents that MEG is most sensitive to (in contrast to radial currents, as would be produced by the crown of the STG). Thus, while the effects seem to localize mainly to the supratemporal plane, we would not draw any strong conclusions along these lines from the data here. On the other hand, as you suggest, the timing of MEG responses is very accurate. Based on this we prefer to talk of different processing stages in the temporal sense. We have added a comment to clarify this in the manuscript (lines 171)

4)	I also wondered about how much the difference in performance between onset STRFs and envelope STRFs might be due to the choice to only have 8 frequency bands in the representation. I wonder might the authors care to wax lyrical on whether or not this result is likely to still be true if one used, say, 100 bands. Might that be enough spectral resolution for us to see these effects also in the envelope STRFs (assuming it was possible to fit the models reliably)?

It turns out that the lack of effect for envelopes was due to generating null models with shuffling (see response to reviewer #1, comment #1). Given what we know now, it is unlikely that more bands would have increased the effect for envelopes, because redundancy between different predictors would have been further increased.

Minor comments:

1)	I wondered why the STRF to the mixture envelope was not plotted in Figure 3 - just for the sake of completeness.

The STRFs shown in Figure 3 of the old version showed STRFs from a model re-fitted after excluding the envelope mixture predictor. The new Figure 3 shows all 6 STRFs.

2)	For people who have not read Bob Carlyon's 2004 paper, I thought it might be nice to slightly unpack the following sentence "Alternatively, the auditory cortex could employ an active process to dynamically recover and represent potential speech features regardless of what stream they belong to, and in doing so provide selective attention with more elaborate representations [11]."

We agree that these ideas are worth expanding upon. In the interest of keeping the Introduction concise we moved the citation from the Introduction to the Discussion at line 414, where the context is now better explained.

3)	The value of the un-masking section on page 16 - and indeed the whole paper really - was brought home to me when I read the line about auditory cortex potentially filling in the underlying speech sources. I thought that was a nice way to think about what kind of thing cortex might be doing in terms of active segregation. I wonder might the authors want to move that point earlier and/or unpack it a small bit for folks who are not familiar (as I happened to be) with references 26 and 27. Just a suggestion.

We agree, and have added more focus on this interpretation of the results to the abstract (line 29) and a brief explanation of the phenomenon for readers not familiar with it (line 261). This point is further emphasized by the new Figure 5.

# Reviewer #4:

This study examines how the foreground and background speech streams are processed when presented simultaneously. Using MEG recordings and linear STRF modeling, it examines how the responses to the onset and envelope of the sources are represented. The results suggest that when the spectro-temporal features mask each other, an active process extracts the onsets of the sources, resulting in a MEG activation that is delayed by 20 ms compared to the activation in response to overt (unmasked) sources. These findings are novel and significant, identifying the brain mechanisms that underlie speech perception in complex situations. It is technically sound and the experiment is well designed. The statistical analysis appropriate and supplementary information is useful. Also, the data needed to replicate the study are available.

There is only one major issue I think needs to be addressed. The authors use linear modeling based on information extracted from onsets and envelops of the signals. A primary conclusion is that onsets are critical for many aspects of the processing. However, the model does not allow too many alternatives to that, as the predictors are only based on onsets or on envelopes. I think the conclusions (including those in the supplement) would be much stronger if offset detectors were built into the model, equivalent to the onset detectors, and if it was shown that no model improvement is obtained from predictors based on the offsets, or, that the offsets themselves cannot predict the results. So, I think it is critical to do additional modeling with the offsets considered before the conclusions of the modeling can be presented as they stand.

This is a great suggestion, and we too would be very interested in including offsets in our investigation. In response to this comment, we reanalyzed the data with onset, envelope and also offset predictors. We summarize the results below. After careful and considered evaluation, we decided that we would prefer not to include the results in the current paper. In short, while the results look promising, and definitely warrant further exploration, we encountered complexities that we believe are beyond the scope of the current paper and would distract from the main conclusions. Importantly, including offsets does not change any of the conclusions we report for onsets or envelopes.

To clarify one point, when saying that onsets are important for stream segregation, we did not mean to imply that other features (like envelopes and offsets) might not be relevant as well. For example, we might predict envelope features of ignored speech to be represented as they are “removed” from the mixture. Even offsets in the background might be tracked, because they indicate when interference in a corresponding spectral band ceases. In the manuscript, we focused on onsets because they signal the beginning of acoustic elements, and would thus be the first evidence, temporally, that the auditory system has for grouping these elements. Grouping decisions about longer lasting sound elements may often be made before processing the offsets. In addition, we focused on onset and envelope representations because those representations have been most commonly used in the past.

Figure R1 (below) shows summarized response functions to onsets and offsets, analogous to Figure 4-E of the main manuscript. We created an offset predictor analogous to the onset predictor, the only difference being inversion of the edge detection receptive field, i.e., to detect negative edges rather than positive ones. The main result was that offsets are significant predictors too, even masked ignored offsets. There was no significant difference between the predictors on the first, positive peak (at ~50 ms). However, on the negative peak (at ~150 ms) there was a significant stream (attended vs ignored) by masking (overt vs masked) interaction. In particular, the negative peak is increased for ignored masked offsets (see Figure R1 below). This result is further confirmation of representations of the ignored speech source, but it is less clear how to interpret the specific pattern. Perhaps masked onsets require additional processing, because they are less obvious?

It appears to us that as such, this result would not add much to the present conclusions. It would, however, increase the crowding on all figures by ~50%. Furthermore, there are strong precedents for using envelope and onset representations, whereas we have less independent motivation for using offsets. Based on this we would prefer not to add the offsets to the manuscript. However, if you think this result should not be omitted, we would certainly consider adding it to the supplementary materials.

Figure R1. **TRFs to onsets and offsets**. From a model with onsets, envelopes and offsets. Details analogous to Figure 4-E.

Minor issues:

L36: in "… an acoustic scene, the acoustic signal …" the word "acoustic" is repetitive. Saying "… an acoustic scene, the signal …" is sufficient.

Fixed.

L507 am -> an

Fixed.
