# Supplementary material for: Neural speech restoration at the cocktail party: Auditory cortex recovers masked speech of both attended and ignored speakers
Source: PLoS Biol. 2020 Oct 22;18(10):e3000883. doi: 10.1371/journal.pbio.3000883 (PMC7644085; doi:10.1371/journal.pbio.3000883)
Supplement: Attachment — Submitted filename: Responses.docx [file pbio.3000883.s009.docx]

Dear Dr. Roberts and Reviewers,

We thank you again for the care taken to evaluate the manuscript, for the thoughtful suggestions, and the overall positive reception. We have extended the manuscript as requested by the editor, and also addressed the remaining points raised by the reviewers as discussed in the point by point responses below.

Sincerely,

Christian Brodbeck, Alex Jiao, L. Elliot Hong & Jonathan Z. Simon

# Editor

IMPORTANT: You'll see that reviewer #2 continues to express deep scepticism about your findings, and there is also a cautious note from reviewer #3. However, after discussing the reviewers' comments with the Academic Editor, we have decided to consider your manuscript further if you make these limitations clear to the readers. Specifically, the Academic Editor would like you to further consider and discuss the issue of the SNR as mentioned by reviewer #2. You should also address the remaining requests from revs #1 and #3.

We thank the editor for the opportunity to address these issues in the final paper. We have extended the discussion of the adequacy and limitations of the auditory model mentioned by Reviewers 2 and 3 (lines 267 ff.) and we also added a short corresponding section on *Limitations* to emphasize the importance of auditory model choices in future research (522 ff.). We also now explicitly acknowledge SNR as an alternative explanation, along with a discussion of why we think it is unlikely to fully explain the results (363 ff., 397 ff.).

# Reviewer #1:

This work by Brodbeck, Jiao, Hong and Simon is a resubmission of a previously reviewed manuscript. The current version of the manuscript addressed most of the concerns of the initial version such that it reads stronger and provides clearer links between results and claims. I very much appreciate the reanalysis and introduction of model fit statistics here with its non-trivial creation of permutated data/predictors as well as the extensive work on the readability of text and figures. However, some comments and unclear points (mainly on the statistics) remained and are listed below.

1.	 Model fit statistics I. Although consistent and significant, the z-values and corresponding r-values for improvement seem to be small and around delta_z = .01 in Fig 2D and otherwise lower than ˜.005 in Figs.3B and 4B. What do you think is the reason for this? Could it be due to correlations between predictors and/or small effect sizes in general? One suggestion might be to indicate the z- or r-value obtained with the tested model for better judgement.

Correlation between variables very likely contributes to the small ∆*z* values, since the ∆ only reflects activity that cannot be explained by any of the other predictors (and some correlation exists inherently between sources and the mixture). More importantly though, we are analyzing un-averaged MEG data in a relatively wide band (1-40 Hz), which means that a considerable part of the “response” signal consists of spontaneous neural activity and non-neural noise that we never hoped to explain. Since the proportion of the data that is “explainable” is thus hard to quantify, we focus on whether an increase in cross-validated model predictions are consistent across subjects, rather than on absolute values. In that sense, an effect sizes based on the distribution of ∆*z* across subjects is closely related to the *t_max_* values we report.

For an estimate of overall model fit, we computed *r* maps in STG, averaged over subjects, and then looked for the maximum across the STG. This was *r* = .143 for the single speaker condition, and *r* = .158 for the two talker condition. We now report these values in the *Methods* section (lines 668 ff.).

2.	Model fit statistics II. I just want to be sure to exactly follow the approach in combination with the source localization. I assume that the dots in Figs 2D, 3B, 4B depict the individual improvements when including a certain predictor vs. not including it and show the average improvement across the x dipole models in each of the hemisphere. When considering the single speaker condition, are these values showing the difference of, for example, the models with onset and envelope vs. models with only the onset to infer the significance of the envelope in this case (this is my guess)? Or do you test the model with envelope vs a null model that is established with a noise simulation (ref 72)?

The reviewer’s guess is correct. We have added the corresponding explanation to the Figure 2 legend. Thank you for pointing out this omission.

3.	l. 326-331. It is investigated whether masked onsets are delayed. However, only descriptive statistics are provided. For better support of the claim, please test for this formally. This comes back in l.382 where it is stated as a summary of masking results that "[these] latency shifts increase at successive stages", which requires a comparison of the latency shifts between earlier and later window.

We apologize that the paragraph was phrased in a way that made the relationship between the results and their statistics unclear. We have rewritten the paragraph to better link the results of the individual comparisons and their corresponding *t*-tests.

4.	Unlike for the t_max it remains unclear to me what the p-values that are mentioned (e.g., ll. 142, 208, …) denote, which is mainly due to the source modeling of signals in each vertex of the modeled brain surface (also in comments on the first version). Are these the corresponding p-values of the statistic of the spatial cluster showing highest significance (as there might be several significant clusters within one hemisphere) or something else? The explanation that "group-level statistics were evaluated with spatial permutation tests" (l. 204) didn't help with better understanding and might benefit from a (very short) description of the procedure outlined in ref 72.

The threshold-free cluster enhancement-based test results in a *p*-value at each vertex, and the reviewer guessed correctly that we report the smallest *p*-value across the brain. We admit that this was not appropriately explained, and have now added the corresponding explanation on lines 679 ff.

5.	Just to be sure this comment is not misunderstood: the following is a final, constructive and, by all means, friendly remark on the topic of masked speech. While re-reading the manuscript and focusing on the masking aspect, I was reminded of Fiedler et al. (2019, NeuroImage) (ref 21). Their results presented in their figure 3 (contrasting relative SNR changes in attended vs ignored speakers) look very similar to the results presented in this manuscripts Figs 4D and E and indicate a similar latency shift (which was not tested for or discussed in Fiedler et al., 2019). It's great and encouraging to see convergence here for different types of data, stimuli and languages (disclaimer: neither am I one of the authors nor am I in any way affiliated with this lab or have any other interests).

Thank you for pointing out this clearly relevant figure, and we have now included the comparison in the appropriate section on line 355.

# Reviewer #2:

My main concern when reading the previous version of the manuscript was that the authors had not supported their primary claim that the cortex "recovers masked speech". Instead, I suggested that the results could be explained by the fact that responses to partially masked onsets (i.e., onsets presented at a low signal-to-noise ratio) would be expected to be lower in amplitude and have a longer latency, based on what we already know about auditory responses to stimuli in noise, from the earliest stages of auditory processing. Indeed, the only justification for calling it "recovery" is that the authors' neural model of onset detection does not detect partially masked onsets in the same way as the measured responses from human cortex seem to do. But this could easily be a problem with their very simple model, rather than any insight into how the auditory cortex processes sound.

The authors now acknowledge this problem, and counter it by providing additional analyses to show that their model (incorporating the overt vs. masked distinction) outperforms a model that incorporates information about the instantaneous target level (quantized into 3 level regions) but ignores the signal-to-noise ratio (SNR). However, this approach does not address the problem in a satisfactory way: The effect of nonlinearity goes beyond just producing level-dependent effects: Nonlinearity produces interactions when two or more stimuli are combined. We already know that at low SNRs (as is the case here, where the average SNR is 0 dB), SNR will modulate the responses to a target sound more strongly than simple intensity. It has nothing to do with "cortical recovery" of signals. Thus, the additional analysis does not rule out, or even reduce, the possibility that what the authors have measured is simply the expected outcome of stimuli that overlap acoustically, producing
partial masking.

In summary, the major potential problem raised in the first round of the reviews seems confirmed: the results are predictable based simply on the fact that neural responses to onsets will be reduced (and slightly delayed) when the onsets are partially masked. This type of response is well established at many stages of the auditory pathways and does not provide evidence for "recovery" or any other type of active cortical processing. Indeed the time course in itself (with latencies of less than 100 ms) should make the authors (and readers) suspicious of the claim that any active recovery process is undertaken.

With this interpretation, most of the speculation provided in the Discussion (Lines 416-494) and the unhelpful black-box model in Fig. 5 become irrelevant. As the data themselves are not new and were already published elsewhere, there seems little else to justify publishing the current study.

As mentioned in the response to the Editor, we now explicitly acknowledge SNR as a potential alternative explanation, followed by a discussion of this interpretation (lines 363 ff. and 397 ff.). We now also acknowledge the potential dependence of the results on the specific stimulus representation, and that we consider this an important point for improvement in the future (lines 267 ff., and *Limitations*).

# Reviewer #3:

Many thanks to the authors for their extensive efforts in addressing my previous comments. I think the new introduction is much clearer. Most of my other comments have been fully addressed also.

I guess I still have a little bit of residual unease about the analysis of masked vs overt onsets. You have determined which onsets are masked and which are not based on your own particular choices of how to represent the acoustic onsets (i.e., using your auditory edge detection model with particular parameters). However, it is not obvious that these onsets will be genuinely masked from the perspective of the cochlear output. If they are not, then should we really be considering them as masked and overt? And if they are, then I still struggle to understand how one can confidently model their separate contributions to the neural responses. I do appreciate that the simulations in Fig. S1 and S2 are aimed to ease our worries on this front – and they achieve that goal mostly. But, I guess, I still feel like we are in a bit of a Catch-22. If they are not perfectly masked, then what does it even mean to call them masked? And, if they are perfectly masked, then how is it possible to separate them at all? If you could help clarify this for me, I would greatly appreciate it.

While our model implements masking as a binary distinction on account of splitting the acoustic source onsets into overt and masked onsets, we are certainly not committed to this being a binary distinction. We prefer to think of features being masked to various degrees. Ideally, we would want to be able to model the response as a function of the degree of masking, but the methods available to us did not allow for this in a straightforward manner. We have now made this consideration explicit in the new *Limitations* section. Consequently, even when features are not perfectly masked, the relevant property for our analysis is that the neural response is not well explained by the overt component of the feature, but it can be better explained when also considering such “hidden”, or masked components (which must be reconstructed/computed since the listener does not have direct access to them).

Also, and I hesitate to suggest this given the amount of work you have already done, but I wondered about the simulations in S1 and S2. Might it not have been more compelling to allow non-zero ignored TRFs and show that there was no warping/delaying of those TRF peaks by the coincidence of the attended and ignored (masked) onsets. Maybe not – the simulation you already have seems fairly compelling. So maybe a general answer to my first comment would suffice.

We are indeed sympathetic to the reviewer’s concerns, and we added this last simulation to the supplementary materials (S1 Simulations, Figure S1C).

# Reviewer #4:

The revised version of the manuscript sufficiently addressed all of my concerns. I do not have any more suggestions.

Thank you for your positive evaluation.
